# A matched-pair study comparing whole-brain irradiation alone to radiosurgery or fractionated stereotactic radiotherapy alone in patients irradiated for up to three brain metastases

**DOI:** 10.1186/s12885-016-2989-3

**Published:** 2017-01-06

**Authors:** Dirk Rades, Stefan Janssen, Liesa Dziggel, Oliver Blanck, Amira Bajrovic, Theo Veninga, Steven E. Schild

**Affiliations:** 1 Department of Radiation Oncology, University of Lubeck, Ratzeburger Allee 160, D-23538 Lubeck, Germany; 2 Medical Practice for Radiotherapy and Radiation Oncology, Hannover, Germany; 3 Saphir Radiosurgery Center Northern Germany, Güstrow, Germany; 4 Department of Radiation Oncology, University Medical Center Eppendorf, Hamburg, Germany; 5 Department of Radiation Oncology, Dr. Bernard Verbeeten Institute, Tilburg, Netherlands; 6 Department of Radiation Oncology, Mayo Clinic Scottsdale, Scottsdale, AZ USA

**Keywords:** Metastases to the brain, Whole-brain irradiation, Radiosurgery, Intracerebral control, Overall survival, Matched-pair study

## Abstract

**Background:**

This matched-pair study was initiated to validate the results of a retrospective study of 186 patients published in 2007 that compared whole-brain irradiation (WBI) alone and radiosurgery (RS) alone for up to three brain metastases.

**Methods:**

One-hundred-fifty-two patients receiving WBI alone for up to three brain metastases were matched with 152 patients treated with RS of fractionated stereotactic radiotherapy (FSRT) alone 1:1 for each of eight factors (age, gender, Eastern Oncology Cooperative Group (ECOG)-performance score, nature of tumor, brain metastases number, extra-cerebral spread, period from cancer detection to irradiation of brain metastases, and recursive partitioning analysis (RPA)-class. Groups were analyzed regarding intracerebral control (IC) and overall survival (OS).

**Results:**

On univariate analysis of IC, type of irradiation did not significantly affect outcomes (*p* = 0.84). On Cox regression, brain metastases number (*p* < 0.001), nature of tumor (*p* < 0.001) and period from cancer detection to irradiation of brain metastases (*p* = 0.013) were significantly associated with IC. On univariate analysis of OS, type of irradiation showed no significant association with outcomes (*p* = 0.63). On multivariate analyses, OS was significantly associated with ECOG performance score (*p* = 0.011), nature of tumor (*p* = 0.035), brain metastases number (*p* = 0.048), extra-cerebral spread (*p* = 0.002) and RPA-class (*p* < 0.001).

**Conclusion:**

In this matched-pair study, RS/FSRT alone was not superior to WBI alone regarding IC and OS. These results can be considered a revision of the findings from our retrospective previous study without matched-pair design, where RS alone resulted in significantly better IC than WBI alone on multivariate analysis.

## Background

Since the prognosis in case of up to three brain metastases is better than in case of four or more lesions, many patients with one to three intracerebral lesions receive stereotactic irradiation or neurosurgery either alone or combined with whole-brain irradiation (WBI) rather than WBI alone [1]. However, since devices for stereotactic irradiation are not available in many centers worldwide, WBI alone is still used also for three or less brain metastases.

Several randomized trials did compare radiosurgery (RS) alone to RS plus WBI [2–5]. These trials demonstrated that RS supplemented with WBI resulted in improved intracerebral control (IC) when compared to RS alone. The benefit not led to improved overall survival (OS). Furthermore, two randomized trials did compare WBI plus a RS boost to WBI alone [6, 7]. One trial was stopped prematurely after inclusion of only 27 patients [6]. According to the second trial, addition of a RS improved control of the irradiated metastatic sites. An OS benefit was seen only for subjects with only one metastasis to the brain.

Until now, only two studies are available that compared RS/FSRT alone to WBI alone. In 2000, a randomized study (*n* = 67) was reported at an international meeting [8]. However, this study has still not been published as a full paper yet. The second study was a retrospective analysis (*n* = 186) from our group [9]. However, this was a retrospective study and distributions of patient characteristics varied up to 7%, which likely caused relevant selection biases. The present study was performed to re-examine our previous results with a new study design that would decrease selection bias. The design of a matched-pair study requiring 1:1 matching of eight factors or each pair of patients was chosen in order to considerably lower the possibility of hidden biases. Furthermore, the current study was conducted in a larger patient cohort than the previous retrospective study (304 vs 186 patients).

## Methods

Three-hundred-and-four patients irradiated for up to three brain metastases (1998 to 2014) were included in this matched-pair analysis. The data were obtained from an existing anonymized database. Ninety patients (39%) were already included in our previous retrospective study [9]. One-hundred-fifty-two patients received WBI alone and were matched regarding eight factors 1:1 to 152 patients treated with RS alone or fractionated stereotactic radiation therapy (FSRT) alone. These were patient age (≤60 vs ≥61 years, median 60 years), gender, Eastern Oncology Cooperative Group (ECOG)-performance score (0–1 vs 2), nature of tumor (breast cancer vs non-small-cell lung cancer (NSCLC) vs small-cell lung cancer (SCLC) vs kidney cancer vs melanoma vs cancer of unknown primary (CUP) vs gastro-intestinal cancers vs gynecological cancers), brain metastases number (1 vs 2–3), extra-cerebral spread (no vs yes), period from cancer detection to irradiation of brain metastases (<15 vs ≥15 months, median period: 14.5 months), and recursive partitioning analysis (RPA)-class (1 vs 2 [10]) (Table 1). These criteria were chosen in accordance with previous studies that identified significant predictors of survival in patients with brain metastases [10–16]. Of those 77 patients of the WBI alone group with extra-cerebral spread at the time they presented with brain metastases, 37 patients (48%) had involvement of one extra-cerebral site (organ), 27 patients (35%) involvement of two sites and 13 patients (17%) involvement of more than two sites, respectively. In the RS/FSRT group, the corresponding numbers of patients were 34 (44%), 27 (35%) and 16 (21%), respectively. The difference between both treatment groups was not significant (*p* = 0.90, chi-square test)

**Table 1:** Distribution of the potential prognostic factors in the two treatment groups

	WBI alone (*n* = 152)	RS/FSRT alone (*n* = 152)
	N (%)	N (%)
Age		
≤ 60 years	78 (51)	78 (51)
> 60 years	74 (49)	74 (49)
Gender		
Female	82 (54)	82 (54)
Male	70 (46)	70 (46)
ECOG performance score		
0–1	104 (68)	104 (68)
2	48 (32)	48 (32)
Nature of tumor		
Breast cancer	31 (20)	31 (20)
Non-small-cell lung cancer	78 (51)	78 (51)
Small-cell lung cancer	4 (3)	4 (3)
Kidney cancer	8 (5)	8 (5)
Melanoma	13 (9)	13 (9)
Cancer of unknown primary	4 (3)	4 (3)
Gastrointestinal cancers	12 (8)	12 (8)
Gynecological cancers	2 (1)	2 (1)
Brain metastases number		
1	86 (57)	86 (57)
2–3	66 (43)	66 (43)
Extra-cerebral spread		
No	75 (49)	75 (49)
Yes	77 (51)	77 (51)
Period from cancer detection to irradiation		
≤ 15 months	76 (50)	76 (50)
> 15 months	76 (50)	76 (50)
RPA-class		
1	59 (39)	59 (39)
2	93 (61)	93 (61)

RPA class three patients were not included, since they are generally considered unsuitable for RS or neurosurgery. Data regarding systemic treatment prior to irradiation were not available, since the database used for this study was anonymized and did not include this information. This applied also to systemic treatment following irradiation.

The patients of this study had up to three metastases to the brain (size ≤4 cm), no previous irradiation or neurosurgery to brain, and confirming of metastatic brain lesions with magnetic resonance imaging. WBI was administered with 6–10 MV photons from a linear accelerator. Fractionation regimens of WBI included 4Gy × 5 (*n* = 122), 3Gy × 10 (*n* = 89) and 2Gy × 20 (*n* = 41). RS was performed with a conventional linear accelerator (*n* = 114), a Cyberknife (*n* = 17) or a GammaKnife (*n* = 23). Of the patients treated with a conventional linear accelerator, 19 patients received FSRT with three fractions of 7 to 12 Gy or five fractions of 5 to 8 Gy. In the patients treated with RS, doses ranged from 15 to 25 Gy (median 20 Gy), which were prescribed to the outer margins of the metastases (75–90% isodose line with linac-based or Cyberknife radiosurgery/FSRT and 50% isodose line with GammaKnife radiosurgery). These doses corresponded to equivalent doses in 2 Gy fractions (EQD2) between 31.3 and 72.9 Gy (median 50.0 Gy) with respect to tumor cell kill (α/β-ratio = 10 Gy). In those patients receiving FSRT, the EQD2 ranged from 29.8 to 66.0 Gy (median 39.4 Gy).

Both treatment groups were retrospectively analyzed with respect to IC (freedom from progression of the irradiated lesions and development of new lesions within the brain) and OS. IC was chosen instead of local control of the treated lesions and freedom from distant metastases, since for many patients of the WBI alone group data regarding the latter two endpoints were not available.

IC and OS were referenced from the last day of irradiation. Intracerebral failure was diagnosed with magnetic resonance imaging (MRI). The exact frequency and number of MRIs following irradiation were not available, since the anonymized database used did not include these data. In general, the follow-up schedule after RS/FSRT included MRI every 3 to 6 months, whereas in most patients receiving WBI MRI was performed only in case of new or progressive symptoms. The univariate analyses of IC and OS were performed with Kaplan-Meier-method supplemented by the log-rank test [17]. After Bonferroni correction (nine tests), *p*-values <0.0056 were regarded significant (alpha-level <0.05). The factors that gained significance or showed a trend (*p* ≤ 0.06) were analyzed multivariate with a Cox-regression-model. Since the RPA-class included age, performance status and extra-cerebral spread, a second analysis was performed if RPA-class and at least one other factor were having significant associations with outcomes on univariate analysis.

## Results

**Fig. 1:**
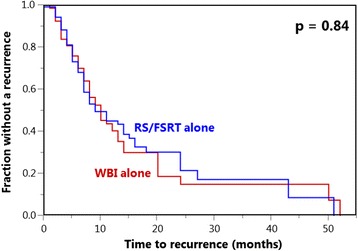
Comparison of whole-brain irradiation (WBI) alone and stereotactic radiosurgery (RS) or fractionated stereotactic radiotherapy (FSRT) alone with respect to intracerebral control (Kaplan-Meier curves)

In the univariate analysis of IC (Table 2), the type of irradiation did not affect outcome (*p* = 0.84, Fig. 1). In contrast, improved IC was significantly affected by single brain metastasis (*p* < 0.001). A trend towards better outcomes was found for a better performance status (ECOG 0–1, *p* = 0.05), favorable nature of tumor (*p* = 0.014) and period from cancer detection to irradiation of the brain metastases of >15 months (*p* = 0.05). The four factors were implemented in a Cox-regression. On multivariate analysis, brain metastases number (risk ratio 1.84; 95%-confidence-interval 1.33–2.56; *p* < 0.001), nature of tumor (1.16; 1.07–1.24; *p* < 0.001) as well as period from cancer detection to irradiation of brain metastases (1.52; 1.09–2:13; *p* = 0.013) were significant, in contrast to performance status (1.28; 0.88–1.82; *p* = 0.19).

**Table 2:** Univariate analysis of intracerebral control

	At 1 year (%)	At 2 years (%)	At 3 years (%)	P
Type of irradiation				
WBI alone	40	15	15	
RS/FSRT alone	45	22	17	0.84
Age				
≤ 60 years	45	20	17	
> 60 years	43	16	16	0.51
Gender				
Female	44	21	18	
Male	42	15	n.a.	0.47
ECOG performance score				
0–1	44	23	20	
2	42	0	0	0.05
Nature of tumor				
Breast cancer	58	28	28	
Non-small-cell lung cancer	46	19	19	
Small-cell lung cancer	50	n.a.	n.a.	
Kidney cancer	39	20	20	
Melanoma	24	9	0	
Cancer of unknown primary	47	0	0	
Gastrointestinal cancers	0	0	0	
Gynecological cancers	0	0	0	0.014
Brain metastases number				
1	50	26	26	
2–3	33	10	8	<0.001
Extra-cerebral spread				
No	44	18	18	
Yes	43	21	14	0.27
Period from cancer detection to irradiation				
≤ 15 months	36	10	10	
> 15 months	50	26	22	0.05
RPA-class				
1	45	21	21	
2	42	17	11	0.07

*n.a.* not available; according to Bonferroni correction, *p*-values <0.0056 were considered significant

Of the 74 patients developing an intracerebral recurrence after RS/FSRT, 39 patients (53%) had an out-field recurrence, 15 patients (20%) and in-field recurrence and 20 patients (27%) a concurrent out- and in-field recurrence, respectively. In this group, intracerebral recurrences were treated with best supportive care alone in 13 patients (18%), WBI alone in 37 patients (50%) and another course of RS/FSRT in 24 patients (32%), respectively. In the 76 patients of WBI alone group developing an intracerebral recurrence, treatment consisted of best supportive care alone in 37 patients (49%), another course of WBI in 30 patients (39%) and RS/FSRT in 9 patients (12%), respectively.

**Fig. 2:**
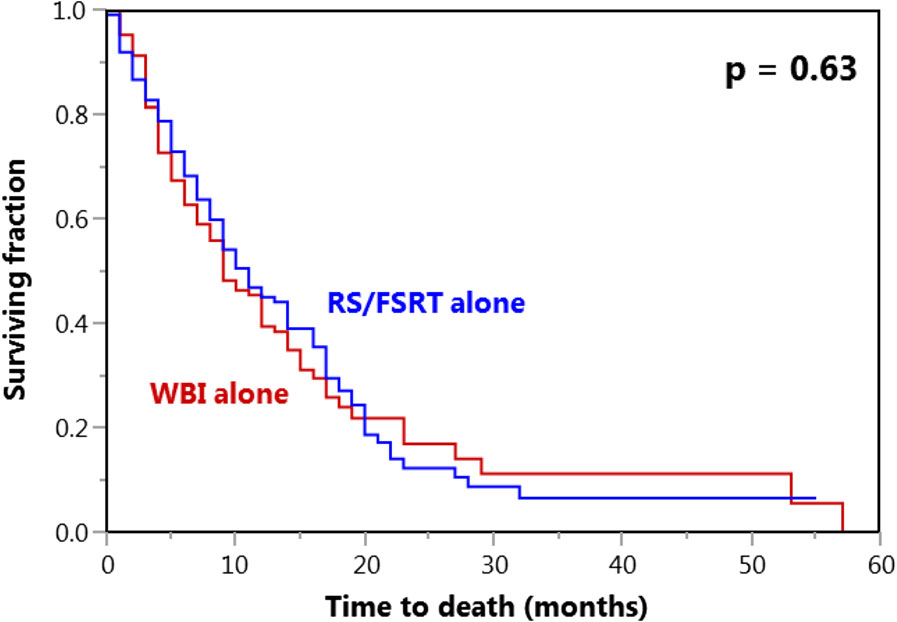
Comparison of whole-brain irradiation (WBI) alone and stereotactic radiosurgery (RS) or fractionated stereotactic radiotherapy (FSRT) alone with respect to overall survival (Kaplan-Meier curves)

Median survival times following irradiation were 10 months in the entire cohort, 9 months after WBI alone and 11 months after RS alone, respectively. On univariate analysis of OS (Table 3), the type of irradiation was not significantly related to outcomes (*p* = 0.63, Fig. 2). In contrast, significantly positive association with OS was shown for ECOG-score 0–1 (*p* < 0.001), favorable nature of tumor (*p* < 0.001), no extra-cerebral spread (*p* < 0.001) and RPA-class 1 (*p* < 0.001). Trends towards improved OS were seen regarding age ≤60 years (*p* = 0.019) or a single intracerebral lesion (*p* = 0.06). On Cox-regression, OS was significantly related to performance status (1.49; 1.10–2.00; *p* = 0.011), nature of tumor (1.07; 1.00–1.13; *p* = 0.035), brain metastases number (1.34; 1.00–1.79; *p* = 0.048), extra-cerebral spread (1.58; 1.19–2.11; *p* = 0.002) and RPA-class (1.84; 1.37–2.50; *p* < 0.001). Age did not show such a relation (1.31; 0.96–1.78; *p* = 0.09).

**Table 3:** Univariate analysis of overall survival

	At 1 year (%)	At 2 years (%)	At 3 years (%)	P
Type of irradiation				
WBI alone	40	17	11	
RS/FSRT alone	45	12	7	0.63
Age				
≤ 60 years	52	17	10	
> 60 years	32	10	7	0.019
Gender				
Female	44	18	13	
Male	41	8	n.a.	0.20
ECOG performance score				
0–1	50	17	12	
2	26	7	2	<0.001
Nature of tumor				
Breast cancer	62	32	19	
Non-small-cell lung cancer	41	11	6	
Small-cell lung cancer	70	n.a.	n.a.	
Kidney cancer	31	21	21	
Melanoma	27	6	n.a.	
Cancer of unknown primary	47	0	0	
Gastrointestinal cancers	28	0	0	
Gynecological cancers	0	0	0	<0.001
Brain metastases number				
1	47	16	10	
2–3	37	12	7	0.06
Extra-cerebral spread				
No	54	17	11	
Yes	31	11	7	<0.001
Period from cancer detection to irradiation				
≤ 15 months	40	10	4	
> 15 months	45	18	14	0.19
RPA-class				
1	61	19	14	
2	31	11	5	<0.001

*n.a*. not available; according to Bonferroni correction, *p*-values <0.0056 were considered significant

## Discussion

Since patients with one to three brain metastases do much better than those with more than three intracerebral lesions, they could benefit from local radiation therapies alone or combined with WBI when compared to WBI alone [1]. However, local therapies such as RS and FSRT are not available in many institutions worldwide, and WBI alone remains the only reasonable non-surgical option. RS and FSRT alone have become more popular during the last years because randomized trials revealed that WBI given prior to RS does not increase the OS in case of one to three brain metastases [2–5]. In these trials, WBI led increased IC-rates but also the risk of radiation-related decline in neuro-cognitive function. Therefore, radiation oncologists are often hesitant to add WBI to RS/FSRT and use RS/FSRT alone instead.

Another issue regarding the irradiation of one to three brain metastases has not yet been answered properly. In case if RS and FSRT are not available for these patients, is WBI alone inferior to RS/FSRT alone regarding IC or OS? Two reports are available that did compare WBI alone and RS alone [8, 9]. A randomized study (*n* = 67) with one to three metastases to the brain, 1-year IC was 62% with WBI alone and 87% with RS alone, respectively (*p*-value not mentioned) [8]. Another study comparing WBI alone to RS alone was a retrospective study of 186 patients [9]. According to its results, better 1-year OS (52% vs 33%, *p* = 0.045) or IC (49% vs 23%, *p* = 0.005) were achieved with RS alone on univariate analyses. On multivariate analyses, IC was significantly different (*p* = 0.003), whereas OS was not (*p* = 0.89). Taking into account the available data for the comparison of WBI alone and RS alone, it becomes obvious that more studies are required that compare these types of irradiation for one to three metastases to the brain. Therefore, it was decided to run a matched-pair analysis including strict matching criteria. The 152 pairs of patients were required to match 1:1 for all eight factors. This design decreased the possibility of selection biases. However, our data are still retrospective and some remaining hidden selection bias cannot be completely excluded. Furthermore, no sufficient data regarding systemic treatment following irradiation were available. A difference between the two treatment groups regarding post-irradiation systemic treatments may have had an impact on OS. In addition, an unknown difference between both groups regarding the frequency and number of post-treatment MRIs might have influenced the IC rates. Since MRIs were generally performed every 3 to 6 months in the RS/FSRT group and only in case of new or progressive symptoms in the WBI group, intracerebral recurrences likely would have been detected earlier in the RS/FSRT group than in the WBI resulting in a false shorter time to an intracerebral failure in the RS/FSRT group. These aspects should be kept in mind when interpreting the results of this study.

RS/FSRT alone did not increase IC or OS rates in comparison to WBI alone up to three years following irradiation. These results are partly contradictory to the findings of our previous retrospective study that did not include a matched-pair design [9]. In that previous study, RS was associated with significantly better IC on multivariate analysis. In a randomized cohort, RS alone provided better IC than WBI alone [8]. However, it is not clear whether the difference was significant, since the corresponding *p*-value was not mentioned in the abstract, and the study is not available as a peer reviewed manuscript. Furthermore, the criteria for inclusion in that randomized study are unknown, which makes it difficult to compare their results to ours. When summarizing the data of the three available studies, it is not clear whether RS/FSRT alone does result in significantly better IC than WBI alone [8, 9]. When considering the largest study, i.e. the present matched-pair study, it appears questionable that a benefit with respect to IC exists for RS/FSRT alone. All three studies agree that RS or FSRT alone do not improve OS when compared to WBI alone. Therefore, if RS and FSRT are not available, WBI alone appears reasonable also for one to three metastases to the brain.

However, neuro-cognitive decline is known to be greater after WBI than after RS/FSRT, mainly due to better hippocampal sparing [3, 5]. In a randomized trial of 58 patients that compared RS alone to RS plus WBI, neuro-cognitive deficits at 4 months following irradiation occurred significantly more common after RS plus WBI than after RS alone (96% vs. 24%, *p* < 0.001) [3]. These results were confirmed in another recent randomized trial of 213 patients [5]. Cognitive deterioration at 3 months following irradiation was observed in 91.7% of patients after RS plus WBI and 63.5% of patients after RS alone, respectively (*p* < 0.001). However, when using modern radiation techniques for WBI such as volumetric modulated arc therapy (VMAT), considerable hippocampal sparing is possible. According to the RTOG 0933 study, hippocampal sparing led to a reduction of neuro-cognitive decline at 3 months following WBI from 30% (historical control) to 7% (*p* < 0.001) [18]. Thus, a randomized trial comparing RS alone to RS plus hippocampal sparing WBI for one to three brain metastases is warranted.

## Conclusion

According to this matched-pair analysis, RS/FSRT alone did not result in significantly better IC and OS rates when compared to WBI alone. These results can be considered a revision of the findings from our previous retrospective study without a matched-pair design, where RS alone resulted in significantly better IC than WBI alone on multivariate analysis.

## Abbreviations

CUP: Cancer of unknown primary
ECOG: Eastern cooperative oncology group
FSRT: Fractionated stereotactic radiation therapy
Gy: Gray
IC: Intracerebral control
OS: Overall survival
RPA: Recursive partitioning analysis
RS: Radiosurgery
